# Particle Tracking and Micromixing Performance Characterization with a Mobile Device

**DOI:** 10.3390/s23249900

**Published:** 2023-12-18

**Authors:** Edisson A. Naula Duchi, Héctor Andrés Betancourt Cervantes, Christian Rodrigo Yañez Espinosa, Ciro A. Rodríguez, Luis E. Garza-Castañon, J. Israel Martínez López

**Affiliations:** 1 Tecnologico de Monterrey, Escuela de Ingeniería y Ciencias, Monterrey 64849, Mexico; a00825462@tec.mx (E.A.N.D.); habc-8@hotmail.com (H.A.B.C.); a01374946@tec.mx (C.R.Y.E.); ciro.rodriguez@tec.mx (C.A.R.); legarza@tec.mx (L.E.G.-C.); 2 Laboratorio Nacional de Manufactura Aditiva y Digital MADiT, Apodaca 64629, Mexico

**Keywords:** micromixers, particle tracking, point of care, microfluidics, open source, mobile application, mixing index, split and recombine (SAR)

## Abstract

Strategies to stir and mix reagents in microfluid devices have evolved concomitantly with advancements in manufacturing techniques and sensing. While there is a large array of reported designs to combine and homogenize liquids, most of the characterization has been focused on setups with two inlets and one outlet. While this configuration is helpful to directly evaluate the effects of features and parameters on the mixing degree, it does not portray the conditions for experiments that involve more than two substances required to be subsequently combined. In this work, we present a mixing characterization methodology based on particle tracking as an alternative to the most common approach to measure homogeneity using the standard deviation of pixel intensities from a grayscale image. The proposed algorithm is implemented on a free and open-source mobile application (MIQUOD) for Android devices, numerically tested on COMSOL Multiphysics, and experimentally tested on a bidimensional split and recombine micromixer and a three-dimensional micromixer with sinusoidal grooves for different Reynolds numbers and geometrical features for samples with fluids seeded with red, blue, and green microparticles. The application uses concentration field data and particle track data to evaluate up to eleven performance metrics. Furthermore, with the insights from the experimental and numerical data, a mixing index for particles (m_p_) is proposed to characterize mixing performance for scenarios with multiple input reagents.

## 1. Introduction

Multiparticle tracking is a crucial quantitative analysis tool for microscopic images for many applications. The ability to obtain images from different types of instruments at different scales has enabled the incorporation of algorithms to monitor and characterize process images for applications such as DNA dynamics [1], cell culture monitoring [2,3], microbiology characterization [4,5], and fluid and sediment velocimetry [6,7].

Microfluidic devices for trapping and sorting cells (and other bioparticles) are ideal candidates for image processing due to the relative ease of obtaining and storing information using conventional light microscopy within the micrometric scale and the interest in monitoring the time-lapse behavior downstream.

Advancements attained in hardware and software enable a new generation of highly integrated sensors capable of processing multiple substances of interest concurrently in real time [8,9]. Beyond the direct progress made in transducer subsystems, efforts have been carried out to increase the efficiency of sensors by engineering the handling of the sample. There is an inherent reagent consumption saving on shifting from macroscale fluid management toward microfluidic-based devices [10]. However, within these operational conditions, the viscous forces become significant, and the laminar flow regime must be considered during the system’s design. Hence, microfluidic device designers have deliberatively incorporated technologies to stir and process samples for the pretreatment of samples in chemical synthesis and analysis, drug discovery, and medical diagnosis.

The mixing process is vital to provide optimal performance for setups where the mass transport of suspended particles is under low Reynolds numbers [11]. Laminar flow is typical for lab-on-a-chip (LoC) and point-of-care (PoC) devices because they operate with channels whose dimensions are under 1 mm. Considering that sample preparation enables subsequential steps for analytical tasks, combining and blending samples efficiently with reagents or other testing agents has a significant impact on the footprint, velocity for processing, and performance of microfluidic devices [12]. Different micromixer designs have been developed and evaluated using multiple metrics such as the length required to achieve mixing, residence time, and mass fraction variance. Machine vision can be used to characterize LoC devices with the parameters mentioned above with high-precision measurements and without human error involved. Furthermore, image-based analysis has the advantage that it can be deployed for high-throughput screening for single-cell and multivariable image analysis [13,14].

Numerous studies have focused on additive manufacturing to produce devices with features below the millifluidic range (0.5–1.0 mm on a feature). The implementation of layer-by-layer manufacturing enables hollow features that have increased the degree of complexity available to manipulate the fluid flows under low Reynolds numbers without the employment of external energy input. These configurations are denominated passive micromixers and rely on increasing the contact area and the contact time between different fluids. Flow can be split, stretched, and folded using periodic structures to achieve homogeneity. Researchers have identified different categories and mixing mechanisms, depending on whether the micromixer design is bidimensional or three-dimensional and whether it includes split-and-recombine (SAR) strategies, patterned grooves, or SAR with two-layer crossing channels. Bayareh et al. have identified a more general taxonomy for passive micromixers, which includes parallel lamination, multilamination, obstacle-based, curved channel, convergence–divergence-based, and asymmetrical configurations [11].

The evaluation of micromixing performance is not a trivial matter. Diffusion or the net movement of molecules from a region of higher to lower concentration ensures that two miscible fluids eventually will be homogeneous with enough time or space. While researchers have reported frequent performances above 90% for active and passive micromixers using measurements based on the intensity profiles, characterizing the degree of mixing over time or footprint restrictions requires the employment of dimensionless numbers such as the Peclet or Reynolds number [15]. Furthermore, with the vast availability of types of micromixing designs and their unique advantages and disadvantages, the comparison among these solutions is not straightforward. While there is a prevalent mixing index (M) in the literature that has been used to characterize the efficiency of microfluidic designs, it has some shortcomings for experimental applications. For example, the index cannot discern a mixing efficiency when there are more than two inlets or when the fluid manipulation requires mixing more than two reagents in a particular sequence.

The characterization of the mixing performance has been mostly underestimated by researchers. There are various techniques to characterize the mixing process, and the applications in which it is applied are in both the macrometric and micrometric scales. In the work of Baqersad et al. [16], a technique based on the frequency histograms of images with linear discriminate analysis (LDA) is used to determine whether the asphalt is segregated or not. Particle mixing in a multiple-spouted bed is studied in the work of Zhang et al. [17] by measuring the tracer particle concentration. The work of Fei et al. [18] presents a method for assessing the uniformity and mixing time of bubbles in a direct contact heat exchanger. This method associates the uniformity coefficient with the local discrepancy of a set of bubbles, which allows a way to characterize the mixture homogeneity and mixing time with the use of a series of images. A comparison of two image analysis methods is carried out in the work of Liu et al. [19] to measure mixing time on rotary drums. The first method uses the concentration variance of the system. The second one, called the contact method, quantifies the mixing quality by counting the interfaces (contacts) of different components on the pixel level. Open-source software (ImageJ, version 1.54f), in the work of Kaspar et al. [20], is used for evaluation of droplet diameter and mixing performance on a microfluidic device. Here, mixing intensity was calculated for every droplet region of interest based on the mean and standard deviation of pixel intensity. In the work of López et al. [21], the mixing efficiency of periodic disturbance mixers is measured based on the ratio of intensity of color on a cross-section of interest and a cross-section reference (beginning of the channel). Recently, a methodology was presented for determining the mixing index based on minimum and maximum red, green, and blue values (RGB) and tested on T- and Y-shaped micromixers [22]. Some of these methods operate at the macrometric scale but can be easily adapted for the micrometric scale with the appropriate process images.

Integral software solutions have been offered on different platforms. For instance, Winer et al. [23] reported the implementation of a particle-tracking algorithm. The authors designed Diatrack, freeware particle-tracking software based on Matlab for particle and cell tracking. However, this software does not provide mixing quantification.

In this work, we present a mixing characterization methodology based on particle-tracking algorithms as an alternative to the commonly used dyes to characterize mixing performance. The presented methodology is based on the open-source software (MIQUOD version 2.0) implemented as an App for Android devices. The open-source software reports measures related to the reduction in the segregation of concentration (mixing index), the reduction in the scale of segregation (mean length scale and index of dispersion), and the potential of change in mixing performance (exposure). The experimental data collected by the proposed method were validated through COMSOL simulations.

## 2. Materials and Methods

### 2.1. Mixing Quantification

Mixing can be defined as the process to combine different substances into a single substance. Mixing is easily performed at the macroscale. However, at the microscale it is challenging to achieve a good mixture because of the laminar flow regime present in most microfluidic systems. Given that, in terms of chemical reactions, a homogeneous mixture is required to achieve better results, there is a demand for novel and effective mixing techniques. Currently, the literature is limited in terms of techniques for characterization of these novel micromixing techniques.

There is no definite way to go from a conceptual definition of a mixture to equations that can quantify the mixture and experiments that directly measure this definition. A quantitative method for expressing the grade of mixing is required and the first proposal can be tracked to the work of Danckwerts [24] and was further discussed by Zwietering [25] in the 1950s. This work suggested that the features of mixing can be defined by the intensity and the scale of segregation. Assuming the mixture as a composition of two mutually soluble liquids and uniform in texture, Danckwerts’ intensity of segregation (I) represents an inverse measure of the effectiveness of the mixing. The following equation defines it as:

(1) $$I=\frac{\sum_{i=1}^{N}{\left(\bar{a}-a_{i}\right)}^{2}}{N\left(\bar{a}\left(1-\bar{a}\right)\right)}=\frac{\sigma_{a}^{2}}{\bar{a}\left(1-\bar{a}\right)}\;,$$

where $a_{i}$ is the concentration of a liquid A at a point in space and an instant in time, $\bar{a}$ is the mean concentration of A in the mixture, N is the number of measurement locations, and $\sigma_{a}^{2}$ is the variance of concentration of A.

For a large 2D data set, the work of Kukukova et al. [26] proposed an alternative approach. According to this work, three measures were directly related to mixing and these are the reduction in the segregation of concentration, the reduction in the scale of segregation, and/or a mixing time scale. These metrics are based on the two statistically defined quantities of Danckwerts and concepts of spatial statistics. They can be evaluated, as shown in [27], using two types of data: concentration field data and particle-tracking data.

#### 2.1.1. Mixing Quantification Using Concentration Field Data

When processing images, the data obtained are an array of pixel colors and intensities. The intensities can be associated with the concentration for evaluating the mixing performance and the concentration field data can be expressed as an m×n matrix of concentration $C_{xy}$ as in Equation (2).

(2) $$\mathrm{Concentration}\;\mathrm{matrix}\;=\left[\begin{array}{cccc} C_{1,1} & C_{1,2} & \cdots & C_{1,n}\\ C_{2,1} & C_{2,2} & \cdots & C_{2,n}\\ \vdots & \vdots & \ddots & \vdots \\ C_{m,1} & C_{m,2} & \cdots & C_{m,n} \end{array}\right]$$

1. Segregation intensity index.

This metric is related to the homogeneity and uniformity. It is commonly quantified by the standard deviation of the concentrations within a target area (σ). In Equation (3), σ is defined relative to the concentration data and the scale of resolution of the measurement, where N is the number of measured points at the target area, $C_{i}$ is the mass fraction measured at the point, and $C_{m}$ is the mean mass fraction of the target area.

(3) $$\sigma =\sqrt{\frac{1}{N}\overset{i=1}{\underset{N}{\sum }}{\left(C_{i}-C_{m}\right)}^{2}}$$

Also, the coefficient of variation (CoV) allows meaningful comparisons between two or more magnitudes of variation, even if they have different means or different scales of measurement. It is defined, in Equation (4), as the ratio of the standard deviation and the mean value. When evaluating concentration profiles in a microchannel with a continuous flow, it is expected that CoV≤1, but there is the possibility of having CoV>1 and it requires the normalization of the CoV by dividing by the initial $CoV_{0}$ at the entrance of the mixer by the value provided by an unmixed condition.

(4) $$CoV=\frac{\sigma }{\mu }=\frac{\sigma }{C_{m}}$$

In this work, the mixing index (M) defined in Equation (5) is based on the coefficient of variation and it is used to define efficiency in micromixers.

(5) M=1−CoV

2. Scale of segregation

The intensity of segregation is not enough to completely define mixing. Therefore, Danckwerts proposed the concept of reduction in the scale to complement the quantification. The scale of segregation evaluates how concentrations are distributed as a function of distance. The correlogram proposed is a plot of the coefficient of correlation of concentration versus the distance separating the data points. Various methods for the calculation of the scale of segregation have been proposed in a wide spectrum of disciplines. Kukukova et al. [28] showed 4 methods to extract length scales from mixing data, but the variogram was selected and it shows the spatial variability or continuity of the underlying concentration data set. The variogram is calculated from:

(6) $$\gamma_{x}\left(h\right)=\frac{1}{2\cdot N\left(h\right)}\underset{N\left(h\right)}{\sum }{\left(C_{is}\left(x\right)-{\;C}_{is}\left(x+h\right)\right)}^{2},$$

(7) $$C_{is}=\frac{C_{i}\left(x\right)-C_{m}}{\sigma \;},$$

where N(h) is the total number of pairs of data separated by a distance h and $C_{is}$ the standarized concentration value at location x. When $\gamma_{x}\left(h\right)=1$, the variability at h has reached the variance of the whole data set and there is no remaining spatial correlation in the data.

3. Exposure

Kukukova’s third performance metric is related to mixing time scale and quantifies the rate of change in segregation. Exposure may be considered as the potential of change in mixing performance. This measure considers the potential to increase mixing regarding contact areas and concentration gradients. E is quantified as:

(8) $$E=\overset{N_{b}}{\underset{j=1}{\sum }}\frac{1}{2}\cdot a_{i,j}\cdot \left|C_{i}-C_{j}\right|$$

where $N_{b}=2,\;3$, or 4 which are the number of neighbors, K=1 is the strength of interaction (analogous to the molecular diffusivity), $a_{i,j}\left\{i,j\right\}=1$ is the contact area per side, and $C_{i}-C_{j}$ is the concentration difference between two consecutive neighbors. Exposure’s definition is a simplified calculation of the rate of mass transfer across an interface.

#### 2.1.2. Mixing Quantification Using Particle-Tracking Data

Particle tracking is the observation of the motion and position of individual particles within a medium. The particle-tracking data refer to a matrix PTV of dimensions n×2, that contains all the positions of the particles and it is defined in Equation (9).

(9) $$PTV=\left[\begin{array}{cc} X_{1} & Y_{1}\\ X_{2} & Y_{2}\\ \vdots & \vdots \\ X_{n} & Y_{n} \end{array}\right]$$

1. Segregation intensity index

To measure homogeneity and uniformity of concentration, CoV is also used for particle-tracking data. Here, concentration profiles are needed rather than positions of particles, so a sampling method is used to extract the concentration data set from particle positions. Kukukova et al. [29] suggested a way of performing the required sampling method based on quadrants for determining CoV. The concentration (C) in a rectangular segment is measured considering Equation (10):

(10) $$C=\frac{P}{A},$$

where P is the number of particles within a square segment of area A. Having in mind that a rectangle area A can be divided in m×n quadrants, Equation (4) can be rewritten as:

(11) $$CoV=\frac{\sigma }{C_{m}}=\frac{\sqrt{\frac{1}{m\cdot n-1}\sum_{q=1}^{m\cdot n}{\left(\frac{P_{quadrant}}{A_{quadrant}}-\frac{P}{A}\right)}^{2}}}{\frac{P}{A}},$$

where $P_{quadrant}$ is the number of particles within a quadrant of area $A_{quadrant}$.

2. Scale of segregation

The maximum striation thickness and the point-to-nearest-neighbor (PNN) distribution methods were selected for processing particle-tracking data. The variance of the PNN distribution is used to evaluate the deviation of the spatial arrangement of particles with respect to the expected homogeneous distribution defined by points of a grid. Kukukova et al. [28] proposed a filtered point–particle variance ($\sigma_{fpp}^{2}$) where the minimum value of the PNN distribution is $X_{R}$ and the distance from each particle is $X_{i}$. The variance is calculated as:

(12) $$\sigma_{fpp}^{2}=\frac{1}{m-1}\overset{m}{\underset{i=1}{\sum }}\left(X_{i}-X_{R}\right)\;\mathrm{where}\;X_{i}=X_{R}\;if\;X_{i}<X_{R}.$$

The work of [30] proposed an index of dispersion ($I_{disp}$) to quantitatively measure the clustering and departure from complete spatial randomness (CSR). The index is the ratio between the variance of the PNN distribution and the mean of the distribution.

### 2.2. Device Characterization

The most common mixing performance metrics include the Reynolds number (*Re*), mixing index, mixing length, and mixing time. The Re is a dimensionless number that measures the ratio of inertial to viscous forces. The Re is an important measure because it allows comparison with other micromixers and its effects on the efficiency of the mixing process. The mixing length is defined as the distance to achieve a full mixing condition and the mixing time is the average time that a particle takes to move from the unmixed position to the fully mixed position [31]. The Re can be calculated by Equation (13):

(13) $$Re=\frac{inertial\;forces}{viscous\;forces}=\frac{LV_{avg}\rho }{\mu }$$

where L is the characteristic length that usually depends on the cross-section and the perimeter of the microchannel, $V_{avg}$ is the average velocity of the flow, ρ is the density of water, and μ is the water viscosity.

The characterization of micromixers can be achieved by analyzing the concentration fluctuations through measurements using transects or target areas in the mixing field. These are usually carried out in simulations. The most common form to characterize mixing performance is by evaluating the intensity profiles in an image obtained through machine vision systems.

### 2.3. Machine Vision

Machine vision can be defined as the use of optical sensing devices to receive and interpret an image of a real scene. This process involves processing an image and carrying out high-precision measurements to be used to perform some activity. Illumination, camera type, and computational power are keys for good performance. Given that digital images must be processed, higher-resolution images require strong computational power; but this also implies an increase in performance. Filters can be used to enhance an image as a pre-processing step, so a balance point must be found between the quality of the camera and the amount of pre-processing applied to the image.

### 2.4. Mixing Quantification Software

The image processing was performed using the custom software MIQUOD (version 2.0) presented in a thesis work [27]; here, the software comprises among other things two libraries. The first library, AForge. Imaging, incorporates the image-processing routines and filters, and the other library, AForge. Video, is a set of libraries for video processing. The source code of the project is available under the terms of GNU (General Public License). MIQUOD is focused on quantifying mixing using machine vision systems and reports measures directly related to the reduction in the segregation of concentration, the reduction in the scale of segregation, and the potential of change in mixing performance. The concentration data type is an array of concentrations associated with pixel intensities and the particle-tracking data are a matrix of particle positions within a target area. MIQUOD processes each type of data separately.

A second version of the custom software (version 1.0) is also programmed in Python. This open-source language is widely used, so a big amount of documentation can be found on different types of platforms. Here, the video and image processing can be achieved with OpenCV Python bindings, a library licensed under two different licenses, 3-clause BSD or Apache 2, depending on the version of the library. These bindings provide a common infrastructure for computer vision applications. This language also allows an easy way to migrate between devices in which the software can be executed.

A third option is programmed in an application for Android devices. Android Studio, the official IDE for Android development, includes everything needed to build Android apps. One programming language for developing apps is Kotlin. It can be interoperable with Java and other languages, which allows an easy way to adapt our previous software. The interfaces (Fragments) for the app were developed with Jetpack Compose which is based in Kotlin. The app is compiled for an API level of at least 30.

#### 2.4.1. Concentration Data

**Figure 1:**
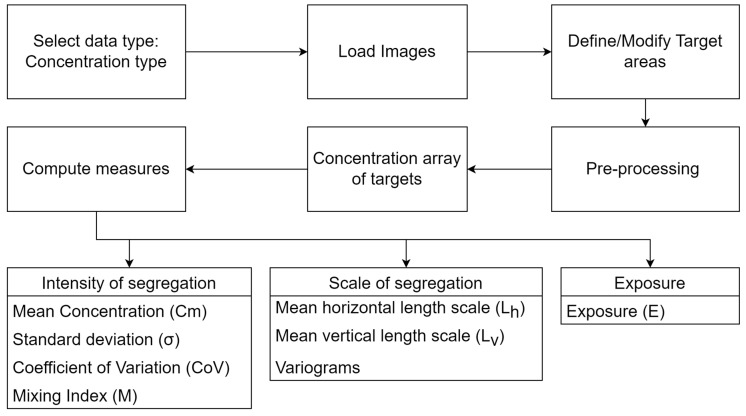
MIQUOD process schematic for processing concentration data type.

The algorithm to calculate the different metrics varies according to the type of data, but that for concentration field data is shown in Figure 1 and follows the following steps:

1. Load image(s) for pre-processing and filtering.
2. Define target areas (areas of interest) to evaluate/quantify without cropping the channel walls.
3. Perform a calibration (optional in the main software).
4. Calculate a concentration array of targets.
5. Compute different measures related to:
  - Intensity of segregation
    - Mean concentration $(C_{m}$)
    - Standard deviation (σ)
    - Coefficient of variation (CoV)
    - Mixing index (M)
  - Scale of segregation
    - Mean horizontal and vertical length scales ($L_{H}$ and $L_{V}$)
    - Horizontal and vertical variograms
  - Exposure (E)

#### 2.4.2. Particle-Tracking Data

**Figure 2:**
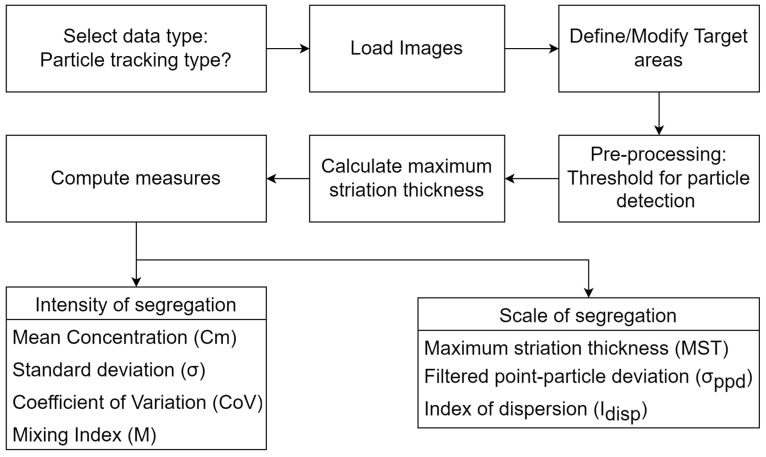
MIQUOD process schematic for processing particle-tracking data type.

For particle-tracking data, the algorithm is similar in most of the steps for concentration field data (Figure 2). The main change is in the maximum striation thickness that is now calculated instead of the concentration array of targets. Also, the measures obtained now are:

- Intensity of segregation
  ○ Mean concentration $\left(C_{m}\right)$
  ○ Standard deviation (σ)
  ○ Coefficient of variation (CoV)
  ○ Mixing index (M)
- Scale of segregation
  ○ Maximum striation thickness
  ○ Filtered point–particle deviation $(\sigma_{ppd}$).
  ○ Index of dispersion ($I_{disp}$)

The work reported by [17] shows a reduction in metrics $\sigma_{fpp}$, $I_{disp}$, and the SR as the particles are more randomly distributed in the patterns, which implies a better mixing process. Resolution of the images can influence the performance metrics. Therefore, it is appropriate to normalize them to provide a type of calibration with respect to an initial measurement (unmixed sample) and to be able to better visualize them in a range from 0 to 1. We propose a mixing index for particles ($m_{p}$) as shown by the following equation:

(14) $$m_{p}=1-\frac{1}{3}\left(\frac{I_{disp}}{{I_{disp}}_{0}}+\frac{SR}{SR_{0}}+\frac{\sigma_{fpp}}{{\sigma_{fpp}}_{0}}\right)\left\{\left(I_{disp},\;SR,\;\sigma_{fpp}\right)\;\in R\;:\;I_{disp}>1,\;SR>0,\;\sigma_{fpp}>0\right\}$$

where ${I_{disp}}_{0}$, $SR_{0}$, and ${\sigma_{fpp}}_{0}$ are the values calculated for the unmixed sample. An index of dispersion ($I_{disp}$) less than 1 indicates a well-dispersed and organized distribution of particles, $I_{disp}\gg 1$ indicates the presence of significant clusters of particles, and $I_{disp}\approx 1$ indicates a good mixing performance is achieved (random distribution). Following this concept and restricted only to $I_{disp}\geq 1$, the proposed metric is equal to 1 when the particles are randomly distributed in the image, indicating a mixed sample. Otherwise, it is equal to 0 which implies an unmixed sample.

### 2.5. Experimentation and Simulation

Testing the performance of the algorithms can be carried out by characterizing either simulations or real experiments as long as an RBG image can be obtained from the process. Simulations were performed first to obtain preliminary results prior to manufacturing the device, so corrections could be made if required. After that, the manufactured ASAR micromixer was tested with dyes and fluorescent microspheres for mixing efficiency and particle-tracking features, respectively. Images were captured from this process and analyzed by the software.

#### 2.5.1. Mixer Designs

Three different micromixer designs are used, as shown in the Results section. The first design is a basic Y intersection with three inlets. The next microdevice considered is a sinusoidal micromixer with helicoidal grooves in the cross-section. The third design is based on a previous design [32] that consists in an asymmetric split and recombine (ASAR) micromixer. The micromixer designs assessed in this paper have been employed in several applications for biosensors and sample processing that employ several mixing stages or multiple inlets [33,34,35]. Furthermore, these micromixer designs were selected to showcase a range of manufacturing complexities.

#### 2.5.2. COMSOL Simulation

**Figure 3:**
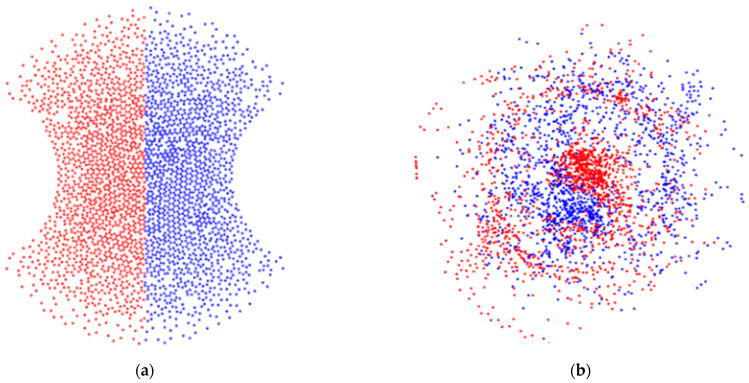
Phase portraits of particle simulation in COMSOL at: (**a**) Inlet of 3D mixer; (**b**) Outlet of 3D mixer.

A finite element numerical simulation was performed in COMSOL Multiphysics software. The modules employed were Laminar Flow, Transport of Diluted Species, and Particle Tracing. The fluid is described by the Navier–Stoke equations and the parameters were set, considering water properties, as follows: density (ρ) was considered as 0$.998\times 10^{2}$ $\mathrm{kg}\cdot {\;m}^{-3}$ and the dynamic viscosity (σ) as $1.01\times 10^{-3}$ $\$\mathrm{kg}\cdot {\;m}^{-1}\cdot {\;s}^{-1}$ according to temperature conditions at 20 °C. Diffusion and concentration coefficients are needed because max flux is given by diffusion and convection. To evaluate the degree of mixing, concentration was set at 10 $\mathrm{mol}\cdot {\;m}^{3}$ in the upper half of the inlet while the lower half was set to zero. The concentration then was assessed at the end of the mixers. The diffusion coefficient was set at $1.24\times 10^{-9}$ $m^{2}\cdot {\;s}^{-1}$. All the parameters were set as reported before in [22]. Figure 3 shows an example of particle-tracking simulation displayed as a phase portrait of the cross-section of the microchannels at the inlet (a) and outlet (b) of the sinusoidal mixer. The figure shows a helical microchannel that has two semicircular grooves.

#### 2.5.3. MIQUOD Experiments

The algorithm was implemented in three different programming languages (C#, Python, Kotlin). Each programming language has its pros and cons. Therefore, we tested three different versions of the software.

### 2.6. Device Manufacturing

To fabricate the microdevices in this work, we used a standard soft lithography process, following these steps:

- First, the mold parts were 3D printed using a Formlabs Form 3 SLA-LF printer (Formlabs, Somerville, MA, USA) with a resolution of 100 microns and Clear V04 material.
- Then, we removed the mold parts from the scaffold platform and cleaned them of uncured resin with isopropyl alcohol. We then post-processed the molds according to the recommended settings: 15 min at 60 °C and 1 h of UV light exposure.
- After that, we mixed the PDMS Sylgard 184 (Dow Corning, Midland, MI, USA) curing agent and polymeric base in a 10:1 ratio and poured it into the assembled mold. We degassed the PDMS in a vacuum chamber and then cured it overnight on a hot plate.
- Then, the cured PDMS slab was detached from the mold using manual tools (a knife and tweezers).
- Finally, we placed the PDMS slab and a microscope glass slide (76 × 52 × 1 mm) into an Expanded Plasma Cleaner (Harrick Plasma, Ithaca, NY, USA) for surface treatment. The plasma removed organic contamination and activated the PDMS surface for glass bonding.

**Figure 4:**
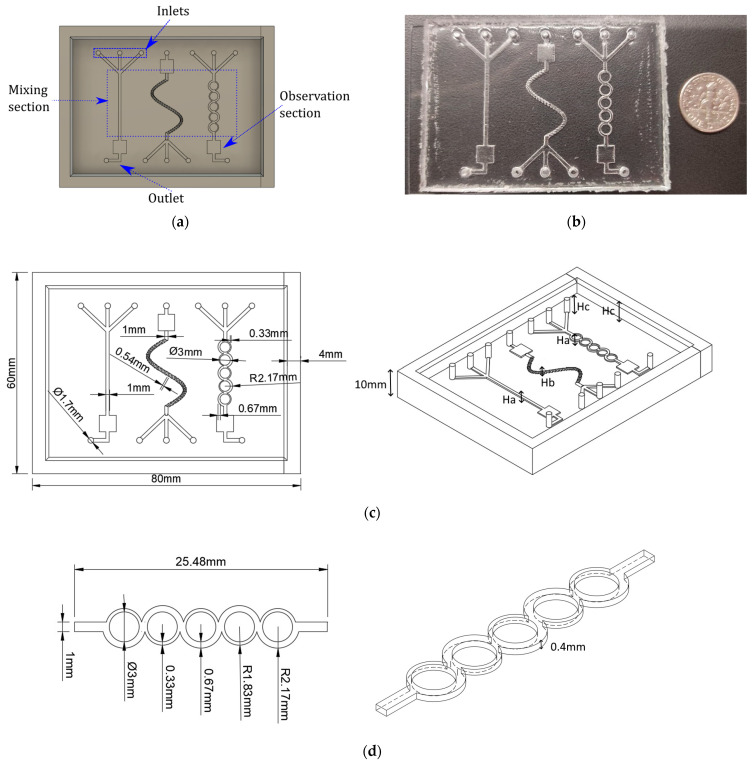
Three inlet microdevices for phosphate concentration monitoring. (**a**) CAD design of the micromixer. (**b**) Manufactured microdevice compared with a dime. (**c**,**d**) Features of the microdevices.

After these steps, we attached the electronic components to the microdevice and made the necessary connections for testing. A CAD, the finished microdevice, and the measurements are shown in Figure 4. More details about the manufacturing can be found in the literature [32,33,36].

The 3D micromixer was inspired by the design presented by Tachibana et al. [37]. That design has a helical microchannel fabricated by lost-wax casting. However, given our manufacturing process capabilities, we followed the procedure presented by Hu et al. [38] to obtain a 3D micromixer based on two 2D micromixers.

## 3. Results

### 3.1. Case Study: Microdevice with Three Different Micromixers

The case study is of a microdevice with different approaches for micromixing three inputs. As shown in Figure 4, it includes: an asymmetric split and recombine (ASAR) micromixer; an observation section after the mixing stage for visualization of the mixture; inlets for sample and reagents and fluid distribution channels. This device was manufactured used the materials and methods previously reported in [33].

The setup for testing the device requires an external NE-1000 programmable syringe pump (New Era Pump Systems Inc., Farmingdale, NY, USA), pipelines, microspheres, and a camera. Three 5 mL syringes were used to pump at the same rate through the inlets of the device. Then, in the observation section, we can capture an image to be analyzed. For the particle detection, fluorescent microspheres (Thermo Fisher Scientific, Waltham, MA, USA) of three different colors were mixed with distilled water. Then, images were taken with a Zeiss inverted microscope (Zeiss, Oberkochen, Baden-Württemberg, Germany) coupled with an apotome system and colibri light panels. The tests were performed at 20× magnification. Following the experimentation by Betancourt [17], the solute–solvent ratio was 10 μL of the fluorescent solutions (F8823; yellow-green 1 μm and F8821; red fluorescent 1 μm) per 1 mL of distilled water. The 14.8 μm fluosphere solution (F8891) had a different concentration of particles per mL. The reagent was used for the experiments without dilution. The Re was estimated by using Equation (13), assuming ρ and μ for water and controlling the flow velocity with the syringe pump.

### 3.2. Android App

**Figure 5:**
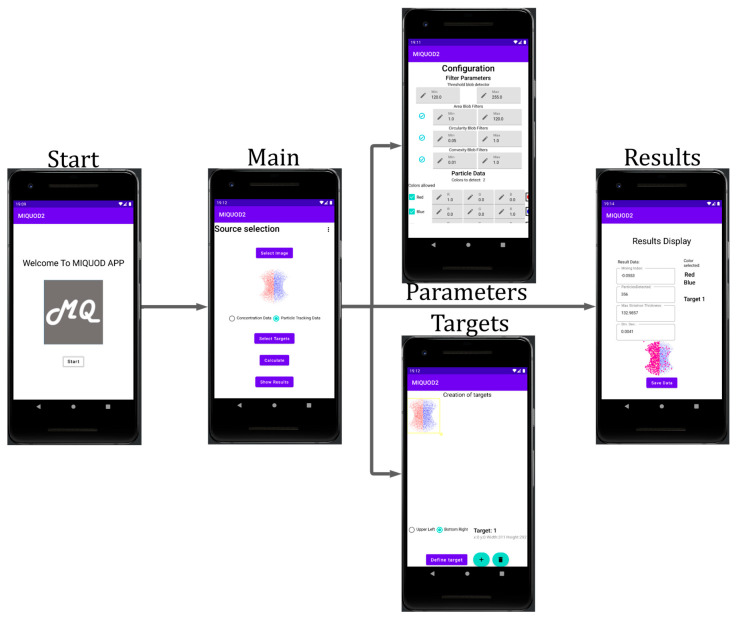
MIQUOD application flow scheme for Android devices.

The Android application was programmed with Kotlin and consists of five interfaces (Fragments) shown in Figure 5. There is an introduction interface with the software name and logo. The second interface facilitates selecting the image source, redirecting to the target selection interface, redirecting to the setup of parameters interface, selecting the type of data to be calculated, and redirecting to the results interface. The third interface allows setting parameters for the filters used in the image-processing interface. The fourth interface sets up selecting the target area in which the calculations are going to be performed. The fifth interface displays the results, including an option to export the data.

The application is compiled for an API level of 30 and tested in an emulated Google Pixel 2. The sequence in which the application can be used is as follows:

1. Select the image source to be processed.
2. Select the target within the image for calculations.
3. Setup or leave as default the filter parameters.
4. Select the type of calculations according to the data.
5. Show the results and export them if required.

### 3.3. Concentration Data

**Figure 6:**
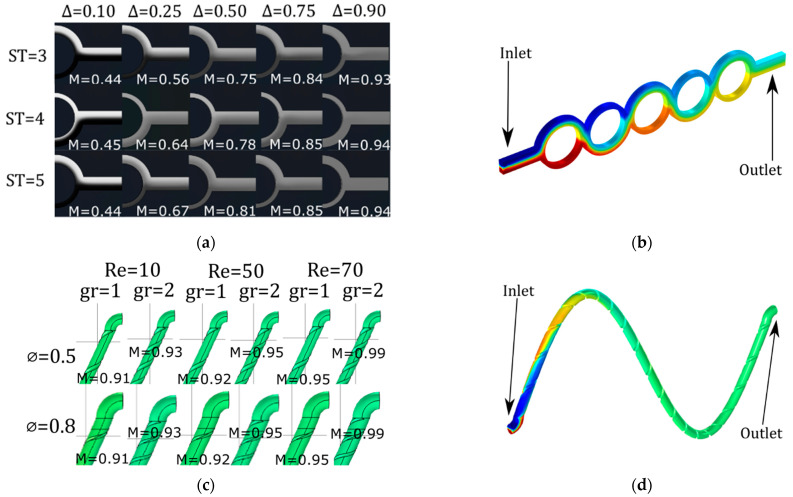
Concentration simulations. (**a**) ASAR micromixer with different stages and ∆; (**b**) ASAR micromixer with 5 stages; (**c**) Sinuosidal mixer with different diamter and grooves downstream; (**d**) Sinusoidal mixer performance example.

The pre-processing stage, as mentioned in Figure 1, of the image for this type of data consists of any external filtering of the image. Not much internal filtering is carried out for this type of calculation. As mentioned in the previous section, numerical simulations in COMSOL Multiphysics were performed to measure the mixing performance to select a design for manufacturing among the variations. Figure 6 shows these simulations: Figure 6a in the outlet for the ASAR micromixer (Figure 6b) with different values of channel depth (Δ in mm) and numbers of stages S at a Reynolds number of 10; Figure 6c in the outlet of the sinusoidal micromixer (Figure 6d) with different numbers of helicoidal grooves (gr), Reynolds numbers (Re), and diameters of the channel (∅ in mm).

### 3.4. Particle-Tracking Data

**Figure 7:**
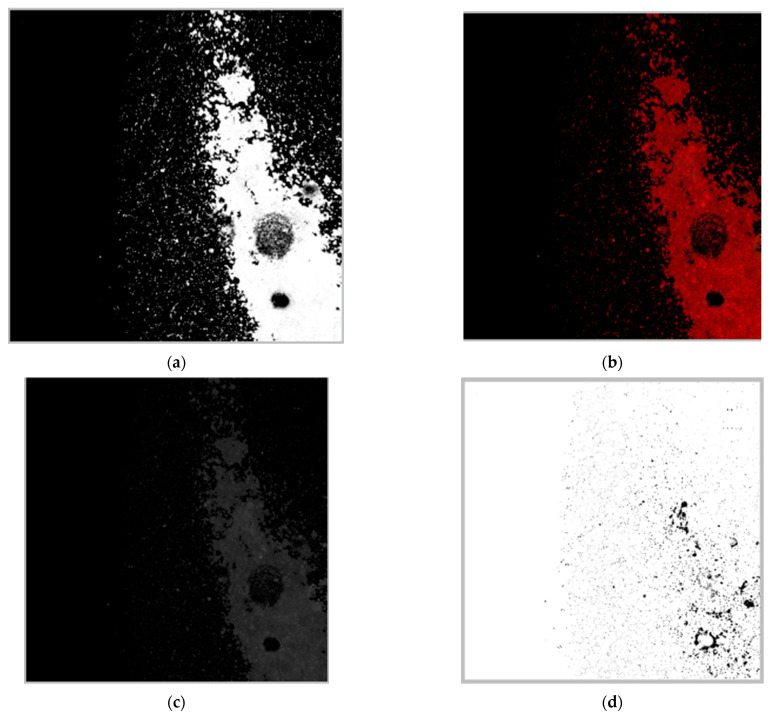
Pre-processing process performed on an image. The stages are: (**a**) Generation of a mask according to the color; (**b**) Bitwise operation with the selection target; (**c**) Gray filtering for simplification of calculations; (**d**) Basic mathematical morphology process (erosion and dilation).

The particle-tracking data first require particle detection in the pre-processing of the image. For this stage, we require several steps: the first converts the RGB color space to HSV color space. Then, we generate a mask according to the color to be detected and filter the image. After that, a mathematical morphological operation is applied to the image and a grayscale filter is applied to simplify the calculations. Finally, blob detection is applied and the particle positions are stored for calculations. This stage is where a difference exists between the three programming languages. Figure 7 shows different stages of the image for the particle detection, then the process is as in Figure 2.

**Figure 8:**
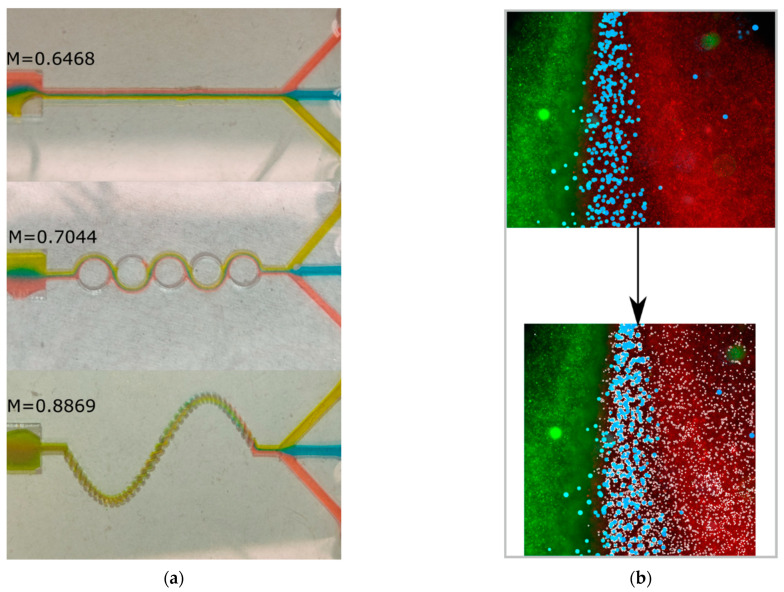
Testing with dyes and particles for the device. (**a**) Dye test in the device; (**b**) Original image and the target selection image with the particle detection feature for red particles (white dots) in the intersection of the inlets.

The test with dyes (Figure 8a) is used to visualize the mixing performance for the case study, but for better tracking of the mixing process we used the fluorescent microspheres and used the MIQUOD software for characterization (Figure 8b). The images were captured at the intersection of the three inlets and at the end of the observation zone of the device. The results of the software are shown in Table 1 for red, blue, and green microspheres.

## 4. Discussion

Figure 8a shows that, among the assessed geometries, the sinusoidal three-dimensional micromixer was the most efficient setup (M = 88.7%), followed by the ASAR micromixer (M = 70%) and the Y-shaped micromixer (M = 64%). The 3D micromixer setup displayed the most uniform distribution at the observation zone. In contrast, for the ASAR micromixer, the dyes are easily discernible by the naked eye. These results agree with the numerical data obtained with the CFD software (COMSOL Multiphysics 5.4) (see Figure 6).

For the particle data, the maximum striation thickness decreases from the input to the observation zone, showing the distance (pixels) of the biggest cluster formed within a striation. Furthermore, the sample downstream has a low filtered point–particle deviation and index of dispersion. Hence, the particles are more dispersed and less aggregated. This behavior is consistent, considering that clusters are broken by the inertial forces and secondary flows induced by the features within the microchannel. Furthermore, the mixed sample displays a higher spatial resolution, meaning that the particles are more evenly distributed. Overall, the data suggest that the mixing process can be characterized by considering how geometrical features of the micromixer influence the dispersion and concentration of particles suspended in fluids in particular regions.

The proposed index ($m_{p}$) was shown to be useful when comparing changes between two states of $\left(I_{\mathrm{disp}},\;\mathrm{SR},{\;\sigma }_{\mathrm{fpp}}\right),$ considering the differences in the image resolutions, number of particles detected, and target area sizes. Table 1 describes the significant changes in the parameters for the red, blue, and green particles before and after being rearranged. These changes could be emphasized by selecting target areas that better comprise the interfaces of the fluids.

For the proposed algorithm, we can assume that, eventually, the particles will be thoroughly mixed ($I_{\mathrm{disp}}\approx 1{\;\mathrm{and}\;m}_{p}\approx 1$). However, beyond this point, a micromixing device can also cause clustering (regardless of whether the sample is properly mixed). For example, Figure 3b shows some accumulation of particles that could be interpreted as poor device performance.

Conventional mixing index determination methods rely on dye-based concentration field data, which are inherently limited by the reliance on pixel intensity measurements. This restriction obstructs the assessment of mixing efficiency for multiple input streams. A particle detection approach, on the other hand, was shown to be effective in characterizing the mixing of multiple inputs. This method, in conjunction with the proposed mixing index, offers a practical solution for evaluating complex mixing scenarios.

MIQUOD serves as a straightforward alternative for assessing dye-based mixing performance. Additionally, the particle-tracking capability provides an enhanced descriptive layer for intricate samples. In this type of detection, color filtering, morphological operations (erosion and dilation), and image resolution play crucial roles in achieving the necessary sensitivity for detecting a substantial number of particles.

The availability of higher-resolution images allows the implementation of cores for the morphological filters (usually multiple iterations of the same filter). In contrast, low-resolution images require smaller operator cores (in some cases, this operation is impossible due to the limited size of the file).

The effective image resolution for particle detection and concentration analysis depends on the specific application. For particle detection, high-resolution images are preferred when the particles are small relative to the pixel size. In these cases, high resolution allows for more accurate identification of individual particles. However, lower-resolution images can be sufficient for detection with particles that are significantly larger than the pixel size. Particles can be detected by identifying blobs within pixels. However, they cannot be detected if multiple particles occupy a single pixel. For concentration analysis, the optimal image resolution depends on the microchannel’s dimensions. While higher-resolution images are generally preferred, there is no specific resolution requirement. Considering that numerical and experimental image acquisition conditions are different, a fair comparison between simulations and experimental data is difficult. Therefore, we present the $m_{p}$ as an alternative to normalize and compare performances.

It should be noted that the MIQUOD software can run with limited computational resources. However, the time required to calculate the parameters may vary, considering the size of the particles, the image resolution, and the number of particles. Depending on the experiment conditions, the Android or PC versions can be more fitting.

## 5. Conclusions

The paper proposes a new method for particle-tracking and micromixing performance characterization. The proposed method is demonstrated via a microdevice with several approaches for mixing three independent inputs. Custom-built open-source software (MIQUOD) was shown to be an effective way to characterize the micromixers’ performance, working with dyes and particles. The sinusoidal 3D micromixer shows the best mixing performance, followed by the ASAR micromixer and the T-shaped micromixer.

The software MIQUOD is effective in characterizing mixing performance when using dyes. However, when operating under a particle-tracking regime, there are additional considerations for a good result. For example, the color pre-processing filter is essential to detect an adequate and significant number of particles.

High-resolution images allow the use of larger cores for the morphological filter and usually require multiple iterations of the same filter. In contrast, low-resolution images require small operator cores, sometimes even restricting the operation to be performed. The relation between the size of the particles and the resolution will determine the number of particles detected and hence the required time. The use of multiple app versions (different languages) allows customization of the results. The proposed index ($m_{p}$) allows a simple way to visualize changes among different micromixer designs and operating conditions.

## Figures and Tables

**Table 1:** Data results for particle tracking from the software MIQUOD.

Mixing Measures		Red		Blue		Green	
		Unmixed	Mixed	Unmixed	Mixed	Unmixed	Mixed
**Intensity of segregation**	σ	0.001347	0.0004542	3.60 × 10^−5^	3.50 × 10^−5^	0.001324	0.0006892
	CoV	1.3517	1.05592	1.504	0.6187	1.4817	1.0252
**Scale of segregation**	MST	447	563	959	1179	370	435
	$\sigma_{\mathrm{fpp}}$	356.1561	239.5713	291.3303	172.25	439.8729	356.1561
	$I_{\mathrm{disp}}$	529.6032	348.1996	282.6921	208.1416	665.691	529.6032
	SR	64.2695	42.2825	292.1343	178.5265	54.631	24.395
	$m_{p}$	0	0.3373252	0	0.353784	0	0.31607
**Particles**	N	5866	2532	141	333	5260	2834

## Data Availability

The software presented in this study is openly available at https://github.com/enaulad511/MIQUOD_Multi.git (accessed on 10 December 2023).
